# Editorial: Advances in Epithelial Ovarian Cancer: Model Systems, Microenvironmental Influences, Therapy, and Origins

**DOI:** 10.3389/fonc.2015.00205

**Published:** 2015-09-22

**Authors:** Viive Maarika Howell, Ben Davidson

**Affiliations:** ^1^Bill Walsh Translational Cancer Research Laboratory, Kolling Institute, Northern Sydney Local Health District, St Leonards, NSW, Australia; ^2^Sydney Medical School Northern, University of Sydney, Sydney, NSW, Australia; ^3^Norwegian Radium Hospital, Oslo University Hospital, Oslo, Norway

**Keywords:** ovarian cancer, mouse models, microenvironment, chemoresistance, ascites, 3D culture models

*Improving outcomes for women with epithelial ovarian cancer is a major health issue worldwide as 5-year survival has not improved significantly over the last two decades*.

The urgent need to increase our understanding of high-grade serous ovarian cancer (HG-SOC) led to it being chosen for the pilot project of The Cancer Genome Atlas (TCGA) and the genomes of over 400 HG-SOC samples are now freely accessible for interrogation. These data are being used for the discovery of biomarkers as well as the generation of hypotheses to understand the natural history of this malignancy and develop effective targeted therapies. In this Research Topic, Lisowaska and colleagues used gene expression profiling to identify that reduced expression of *CLASP1*, a regulator of microtubule dynamics essential for mitotic cell cycling was positively associated with survival, either overall or disease free (1). Their data clearly highlighted that different histological subtypes of ovarian cancer had very different molecular signatures, although undifferentiated and high-grade serous ones were indistinguishable. These results are consistent with other findings that together have led to the understanding that epithelial ovarian cancer (EOC) is not a single entity but rather a number of distinct malignancies with different etiologies and molecular aberrations. Of these, HG-SOC is the most aggressive and common, accounting for 60–70% of all cases of ovarian cancer.

Notwithstanding the unprecedented *in silico* resource now available via TCGA and other web-based portals, research in HG-SOC is hampered on a number of fronts. Comparison of results from cancerous or cancer-associated stromal cells with each non-cancerous (normal) equivalent is a fundamental research question, but what to use for normal cells is unclear. Recent evidence suggests that HG-SOC has its origins in the secretory cells located in the fimbrial end of the fallopian tube. This is contrary to the prevailing notion that HG-SOC arises from the epithelium lining the ovary and inclusion cysts. The contribution of each site to serous ovarian carcinogenesis is currently under debate. Jones and Drapkin recount the evolution of evidence for each site of origin and review the use and limitations of primary cell culture model systems developed from each site (2). In support of an ovarian surface epithelial (OSE) origin for HG-SOC, McCloskey and colleagues describe the tumor-initiating characteristics of a novel spontaneously transformed mouse OSE cell line (3). Ahmed and Stenvers draw attention to ascites, an underutilized yet readily accessible source of primary cancer cells from EOC patients and provide a detailed review of its use as a clinically relevant model system (4). Different methods of isolation and culture of primary ovarian cancer tumor cells from ascites and other sources are reviewed by Cunnea and Stronach who also advocate for universal standardized protocols for improved reproducibility and interpretation of results between studies (5).

In conjunction with the development of new cell models is the move to three-dimensional (3D) culture conditions. 3D culture systems feature in several articles in this Research Topic indicating the enthusiasm for this culture type and recognition of the deficiencies of the monolayer systems (2, 5, 6). It is hoped that models with more physiologically relevant microenvironments will lead to increased clinical translation of findings. 2D and 3D culture systems are compared by Fuller and Howell and the characteristics of the different matrices available reviewed (6).

Resistance to platinum therapy continues to be a major issue in HG-SOC (5, 7, 8). Cunnea and Stronach review the currently available immortalized cell lines in the context of sensitivity and resistance to platinum and highlight deficiencies in *in vitro* development of treatment resistance (5). In particular, they illustrate the power of having serial cell lines from the same patient which have been able to show that the development of treatment resistance was due to the selection of resistant sub-clones rather than evolution of new lineages. They note the need for more such matched cell lines, which is now one of the goals of the European OCTIPS (Ovarian cancer therapy – innovative models prolong survival) consortium.

In light of the inevitable development of platinum resistant or refractory disease (7), new strategies and agents to reduce tumor burden and improve patient outcomes are in high demand. Essential for preclinical development are accurate and robust EOC model systems. The current status of EOC models specifically for preclinical development is reviewed by Konstantinopoulos and Matulonis (9). Different theories for overcoming platinum resistance are detailed in this Research Topic. Evidence from genetic and functional studies of HG-SOC point to the homologous DNA repair (HR) system, in particular BRCA1 and BRCA2, as critical determinants of response to platinum therapy. Wiedemeyer and colleagues discuss the concept of disrupting HR capacity via BRCA1/2 to reverse platinum resistance in BRCA-proficient cancers (8). They provide a comprehensive overview of available agents and the genetic aberrations targeted by these agents illustrating how rational therapeutic combinations may be designed to prevent or delay the onset of platinum resistance in HG-SOC. Alternative theories of platinum resistance are proposed by Chien and colleagues that also take into account the paradoxical recurrence of platinum sensitivity observed in patients with HG-SOC (7). They present the cancer stem cell theory for both the development of resistance and sensitivity upon recurrence and highlight the involvement of specific components of the extracellular matrix in the establishment of stem cell niches. Based on this evidence, they suggest that cancer cells are not intrinsically resistant to platinum but, rather, acquire extracellular matrix-dependent platinum resistance.

Targeting cancer stem cells is the basis of a new therapy identified by Abubaker and colleagues (10). They observed that paclitaxel treatment enhanced the expression of cancer stem cell-like markers in surviving cancer cells *in vivo* and coincided with significant activation of the JAK2/STAT3 pathway. In their research manuscript, they report the efficacy of concurrent paclitaxel and JAK2/STAT3 pathway inhibitor, CYT387, in a xenograft model of HG-SOC.

Having a familial *BRCA1/2* mutation remains the best defined risk factor for HG-SOC. The search for HG-SOC precursor lesions led to the discovery of P53 signatures, serous tubal intraepithelial lesion (STILs) and serous tubal intraepithelial carcinomas (STICs) all in the fallopian tubal epithelium, implicating this tissue as a site of origin for HG-SOC. George and Shaw provide a critical review of the literature related to these findings, noting that P53 signatures are found with similar frequencies in *BRCA*-mutation carriers and non-carriers, and that STILs and STICS are uncommon and identified with poor reproducibility (11). They review what is known regarding the involvement of hormones in HG-SOC development with specific reference to the altered reproductive physiology in *BRCA*-mutation carriers and propose that the combination of ovulation-induced chronic inflammation coupled with *BRCA*-mutations may predispose the development of precancerous lesions leading to HG-SOC.

While the changing notion of the site of origin of HG-SOC is critical for understanding the molecular mechanisms of ovarian cancer, it also has “real world” implications for prophylactic strategies for women with familial *BRCA* mutations or a strong family history of breast and ovarian cancer. Shenenberg and Mitchell review the molecular evidence providing rationale for risk-reducing bilateral salpingo-oophorectomy *versus* bilateral salpingectomy *versus* bilateral salpingectomy and delayed bilateral oophorectomy (12). This is elegantly balanced against quality of life issues that each individual faces when determining which risk-reducing procedure if any, is most appropriate for them. Non-surgical alternatives that may reduce risk and advances in diagnostic imaging for improved early detection, including current research assessing folate receptor α and HER-2 for tumor-specific imaging are reviewed by Ohman and colleagues (13).

Understanding disease development requires models of tumor progression and is best addressed by spontaneous mouse models. However, there is a paucity of reproducible spontaneous animal models of HG-SOC. This may be partly explained by the lack of promoters to drive genetic changes in the cell of origin of this disease. However, circumventing this technical difficulty by surgical delivery of Cre recombinase has not always delivered the expected outcomes. Does this again relate to the cell of origin? New models for the study of HG-SOC are being generated to accommodate the changing view of the cell of origin and enable monitoring of tumor progression. House and colleagues and others provide overviews of the results of using different methods to direct genetic changes to the OSE and FTE (2, 13, 14). Smith and colleagues discuss the importance of including menopausal physiology in mouse models of EOC and propose the germ cell-deficient Wv mice (*c-kit* mutant) as a background strain for breeding with relevant genetically engineered changes (15).

While grafted models of HG-SOC are not suitable for assessing early stage disease, their faster time course and the ability to easily add in reporters for *in vivo* imaging provide advantages over the spontaneous models. House and colleagues review the few reported syngeneic models of HG-SOC as well as the imaging modalities available for *in vivo* studies (14). The characterization of an FVB/N strain syngeneic model of HG-SOC by McCloskey and colleagues, reported for the first time in this Research Topic, thus has the potential to be an exciting and useful addition to the armory of reagents available for ovarian cancer research (3).

Xenografts are the most utilized *in vivo* platform for many cancers, including EOC. However, it is well recognized that they have deficiencies in recapitulating the tumors they represent. These include the loss of fidelity through *in vitro* culture, lack of cellular heterogeneity and lack of an intact immune system. The advent of patient-derived xenograft (PDX) models was aimed at overcoming two of these deficiencies by using pieces of patient tumors that had never been cultured *in vitro* and that retained the microenvironment of the original tumor. PDX models are aimed primarily at testing drug responses. The site of implantation of PDXs can alter the rate of engraftment as well as the characteristics of the model. The advantages and disadvantages of the different sites of implantation are discussed by Scott and colleagues as part of their review of PDX model systems that have been trialed for EOC (16). They and others also assess the utility of PDX models as preclinical models for trialing new therapies for EOC (2, 13, 14, 16).

The rapid acceptance of PDXs as preclinical models underscores the increasing awareness of the contribution of the microenvironment to the pathogenesis and progression of cancer. An overview of the different components of the microenvironment such as proteases and extracellular matrix and their roles in promoting invasion and metastasis in EOC is presented by Davidson and colleagues (17). Dissemination of this cancer by shedding of cells into the peritoneum is a major route of metastases and involves interactions between cancer cells and adipocytes, endothelial cells, fibroblasts, mesenchymal stem cells, macrophages, and other immune cells. The microenvironment of the peritoneal cavity that makes it highly conducive to carcinomatosis and the therapeutic implications of targeting the heterotypic cellular interactions within the peritoneum are the focus of the review by Naora (18). Within the immune population, tumor-associated macrophages represent the most abundant infiltrating immune cell in human ovarian tumors and ascites. They display a unique activation profile in ovarian tumors and are able to create an immunosuppressive microenvironment, allowing tumors to evade immune detection and promoting tumor progression. This cell type is the focus of mini-review by Colvin (19).

In summary, this Research Topic showcases our current understanding of a number of key areas in EOC. It has a strong focus on model systems given the critical importance of having accurate systems and the particular difficulties and dilemmas faced especially in the development of *in vivo* models.

## Conflict of Interest Statement

The authors declare that the research was conducted in the absence of any commercial or financial relationships that could be construed as a potential conflict of interest.
